# Concept(s) of Health: Lifestyle at the Heart of Modern Health

**DOI:** 10.1007/s10670-025-00971-3

**Published:** 2025-06-25

**Authors:** Kevin Reuter, Andrew J. Latham, Somogy Varga

**Affiliations:** 1 Department of Philosophy, Linguistics and Theory of Science, University of Gothenburg, Renströmsgatan 6, 41256 Gothenburg, Sweden; 2 Department of Philosophy and History of Ideas, Aarhus University, Aarhus, Denmark; 3 CPEMPH Research Associate, University of Johannesburg, Johannesburg, South Africa

## Abstract

One significant project within the philosophy of medicine has been to explore the concepts of health and disease and their conceptual relationship—whether health is the absence of disease (negativism) or the presence of a positive state or capacity (positivism). While some contend that this project is hampered by some limitations of traditional philosophical analysis, this paper employs a multi-method approach, incorporating corpus linguistics, semantic feature production tasks, and vignette studies, to investigate the concept of health. Our principal finding is that adherence to healthy behaviors is one of the key criteria for assessing overall health. This finding not only contributes to the philosophical-medical debate but also has implications for health communication and policy development.

## Introduction

Over the past five decades, one major research project in the philosophy of medicine has been to provide an account of the concepts of health and disease using the methods of conceptual analysis (for reviews, see e.g., Murphy, 2021; Kingma, 2019; Giroux, 2016). Within that research project, one core issue stands out: how health and disease are conceptually related to each other. There are two major positions in the literature: *negativism*, which claims that health is the absence of disease (e.g., Boorse, 1977, 2014; Wakefield, 2014) and *positivism*, which holds that health is the presence of some independent positive state or ability (e.g., Nordenfelt, 2017; Tengland, 2020; Venkatapuram, 2013; Wren-Lewis & Alexandrova, 2021).

The project of providing an account of our concepts of health and disease and the relationship between them has faced two major challenges, linked to the methodological limitations of conceptual analysis. First, critics have pointed out that debates between the aforementioned positions have become entrenched, with traditional methods, such as the method of cases, toggling indecisively between competing intuitions (e.g., Fuller, 2018; Lemoine, 2013; Schwartz, 2017; Sholl, 2015). Second, some critics contend that this research project has not sufficiently attended to the rapid changes that have occurred to the concepts of health and disease (Cooper, 2020). What constitutes health and disease is “on the move”, as Rachel Cooper (2020) puts it. Further, the rapid accumulation of facts about the natural world has also resulted in rapid changes in what cases are intuitively thought of as being cases of health and disease.

Recently, some theorists have suggested that in order to make progress, we should supplement traditional methods with empirical approaches (e.g., Aftab, 2021; De Block & Hens, 2021; Faucher, 2021; Hens & De Block, 2023; Lemoine & Okholm, 2023; Machery, 2023). Indeed, for our purposes, we assume that adopting such methods might go some of the way to addressing the two challenges we have just described.

First, empirical data can be used to help identify those accounts that better align with the ordinary understanding of health and disease—something that is viewed as a desirable feature by many theorists (e.g., Wakefield, 1992, 2014; Cooper, 2002; Nordenfelt, 2017). Of course, our approach and findings will be most directly relevant for those who adopt *unrestricted negativism* (i.e., the view that negativism is true of both the professional and lay concept of health, as proposed by scholars like Wakefield, 1992, 2007; Cooper, 2002),^1^ and perhaps less so for those embracing *restricted negativism* (i.e., the view that negativism is true of the concept of health which is deployed in theoretical medicine, as proposed by scholars like Boorse, 1997, 2014) or those engaged in revisionary approaches (e.g. Griffiths & Matthewson, 2018; Walker & Rogers, 2018). However, there is some relevance for views beyond unrestricted negativism. Although our study primarily focuses on the lay concept of health and does not explore how health is understood within scientific or professional circles, drawing on Dominic Murphy (2021), we recognize that health and disease are not confined to scientific or common-sense realms but intersect both. This intersection imposes limitations on how significantly scientific or expert concepts can diverge from the lay concept, meaning that expert concepts should maintain some alignment with common usage to remain relevant and recognizable as health concepts. Moreover, even revisionary or “engineering” projects should find the empirical data useful, as they are justified based on the descriptive accuracy of existing concepts, and grounding them in experimental philosophy could help adhere to the constraints set by commonsense usage (Machery, 2023). Second, empirical data, especially corpus-linguistic data, can highlight historical shifts in the concepts of health and disease, which might be used to model future changes. Recent research demonstrates that significant semantic shifts, as seen in terms like “gay” and “awful”, can be reliably tracked using corpus-linguistic methods (Simpson et al., 1989; Hamilton et al., 2016).

In the current paper, we will present a range of new empirical data from corpus linguistics, semantic feature production tasks, and vignette studies on the concept of health.^2^ Our primary focus will be the relational issue: that is, whether health is conceptualized merely as the absence of disease or the presence of something more. Some have noted that health should be seen as something more, because it can coexist with at least some diseases and disabilities (Carel, 2016; Venkatapuram, 2013). Traditionally, this “something more” has been linked in the literature to the presence of some sort of well-being (e.g., World Health Organization, for a discussion, see Schramme, 2023) or to certain capacities or resilience that promote some level of well-being (e.g., Nordenfelt, 2017; Graham, 2010; Keller, 2020). However, earlier evidence suggests that it may also include health-related behaviors commonly grouped under the umbrella of “lifestyle”. Indeed, some studies indicate that health, for some, extends beyond the absence of disease and is associated with the presence of a healthy lifestyle (e.g., Blaxter, 1990, 2010). This perspective is increasingly relevant, considering advances in medical science highlight the significant impact that lifestyle choices have on health outcomes—a notion that has been amplified by public health messaging. This purported link between health and lifestyle is what much of our paper aims to explore.

The paper is structured as follows. Section 2 presents the results of our corpus-linguistic study. There we observe a shift in the usage of the term “healthy” away from indicating the absence of disease and towards lifestyle-related matters. Recognizing the limitations of corpus analysis, Section 3 further investigates this emerging association using Semantic Feature Production Tasks. In Section 3.1, we explore the influence of lifestyle on people’s health assessments across several experimental tasks. Finally, Section 4 presents a general discussion and outlines the implications of our findings.

## From Healthy Individuals to Healthy Lifestyle: A Corpus Study

The rise of corpus linguistic techniques has sparked an increased interest in exploring conceptual questions using extensive language datasets (Reuter et al., 2023; Reuter & Baumgartner, 2024; Willemsen et al., 2023).^3^ By examining large collections of actual language usage, corpus linguistics offers a window into the real-world application of words and ideas, serving as an empirical tool for understanding language use across varied situations. Additionally, tools like the Corpus of Historical American English (COHA) and Google’s Ngram Viewer enable researchers to trace language changes and understand shifts in the meaning of terms.

### Corpus-Linguistic Analyses

A central step in our investigation into the use of “healthy” involves examining how the adjective “healthy” has been applied throughout the past two centuries. By inputting “healthy NOUN” into the COHA search field, we obtain a dataset revealing the nouns most frequently conjoined with “healthy” during the last 200 years. Table 1 (left-hand side) presents the nine most prevalent nouns, highlighting that terms related to or denoting individuals are the predominant nouns featured in this corpus. These results should not be surprising, as the term “healthy” naturally pertains to individuals. Similar findings can be achieved by examining the most frequent nouns paired with “healthy” using the Ngram Viewer. With two exceptions, the usage of these combined phrases has remained relatively stable over the past 200 years. First, we find a decrease in the use of “healthy man” and an increase in the use of “healthy person”. This effect can be readily attributed to the shift from “man”, once used in a gender-neutral context, to the more explicitly inclusive term “person”. Second, the phrase “healthy body” has been far more popular during the last 30 years than before.

**Table 1:**
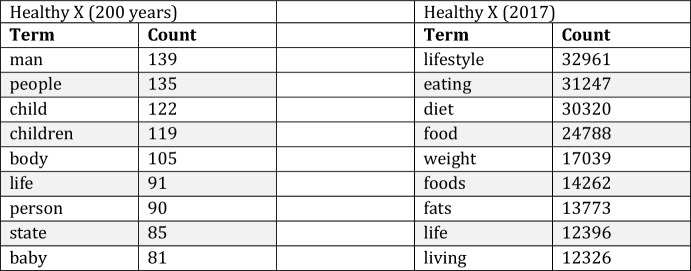
The 9 most frequent nouns occurring after “healthy” over 200 years (left, COHA) and in 2017 (right, iWeb)

**Fig. 1:**
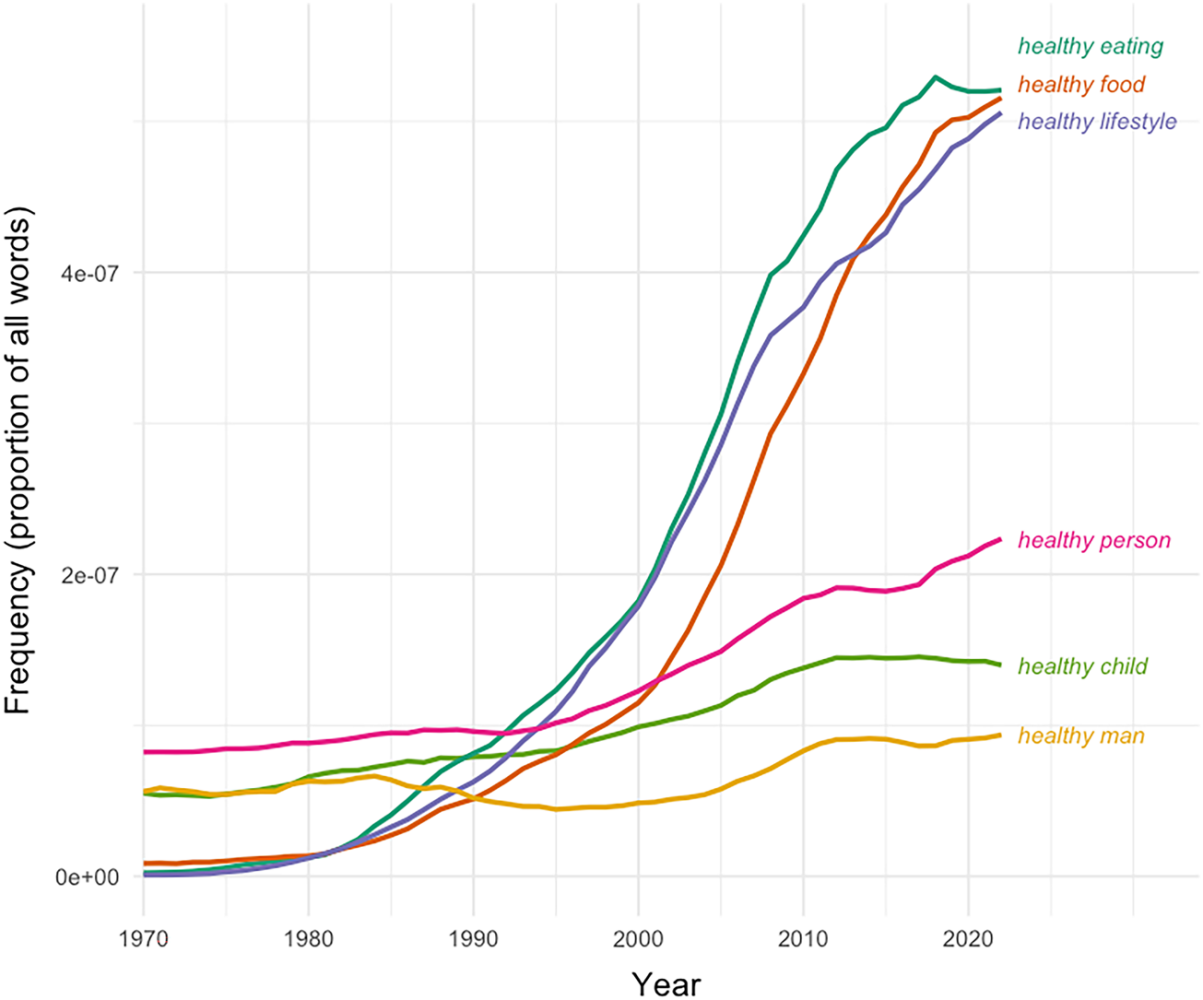
Development of the frequency of nouns conjoined with “healthy” using the Ngram Viewer

A very different picture unfolds when we examine the *contemporary* use of the term “healthy”. Utilizing the iWeb Corpus, one of the largest systematic freely accessible corpora, the same search term reveals that the nouns most frequently associated with “healthy” have significantly changed. Previously, the usage predominantly referred to individuals, but by 2017, the most common nouns had shifted to include “lifestyle”, “eating”, “diet”, “food”, and “weight” (see Table 1, right-hand side). To determine whether these terms have always been in use or whether there has been a notable increase in recent decades, we again turn to the Google Ngram Viewer, entering three of the most frequent nouns identified in the iWeb corpus. For all three, the year 1980 emerges as a pivotal moment, marking the start of a trend where their usage significantly increased. (see Fig. 1). This data from the corpus signals a dramatic shift in how “healthy” is applied in everyday language. Additionally, these lifestyle-related terms are mentioned with “healthy” two to three times more often than nouns that refer to individuals, underscoring the change in focus.^4^

The predominant association of the term “healthy” with lifestyle-related matters such as eating, food, and diet since the 1980s can (in part) be attributed to the influence of a number of factors. The rise in health consciousness, introduction of dietary guidelines, fitness and wellness movement, and so on, have all collectively shifted the focus of health toward preventive medicine and the importance of diet and exercise (Biddle & Mutrie, 2008; Committee, 2015; McGinnis & Foege, 1993). Similarly, lifestyle matters such as smoking, alcohol intake, poor dietary habits, and insufficient activity, have become prevalent in the medical literature. That is because lifestyle is recognized as having a significant influence on the development and progression of diseases and health outcomes (Brivio et al., 2023; Chou et al., 2009; Thirlaway & Upton, 2009; Willett, 2012). It is also very likely that this shift in association has been further supported by the food industry’s strategic marketing, which has capitalized on the growing demand for healthier options by promoting products as being components of a “healthy lifestyle” (Nestle, 2013). Each of these developments has helped to contribute to ingraining “healthy lifestyle” practices into public consciousness.

### Corpus-Linguistic Data and Past Experimental Work

Despite the corpus data showing a remarkable shift in the use of “healthy” (and arguably other health-related vocabulary), it would be premature to conclude that the concept of health itself has changed. The rise in lifestyle-related vocabulary may simply reflect an increased understanding of the causal relationship between lifestyle choices and health. Let us illustrate this with a health-related example. Over the past few decades, our knowledge about cancer has expanded significantly, encompassing its genetic basis, the role of carcinogens, and the mechanisms of metastasis. However, irrespective of these advances, the term “cancer” still fundamentally refers to a group of diseases characterized by the uncontrolled division and spread of abnormal cells. Indeed, while the literature on health-related lifestyles has proliferated, few scholars have seriously considered that the meaning of health is now closely tied to lifestyle choices. One notable exception is Mildred Blaxter, who analyzed data from the Health and Lifestyle Survey (Cox et al., 1987), which included a substantial sample size of over 9000 participants. This survey not only assessed individuals’ fitness and well-being but also included several questions about how participants themselves understood the term “health”. When asked the question, “Think of someone you know who is very healthy. What makes you call them healthy?”, 23% of adults aged 18–39, 16% of adults aged 40–59, and 12% of adults over 60 indicated that a healthy lifestyle was central to health. Blaxter (1990, 23; see also Blaxter, 2010) notes, “For these respondents, health was *identical* [emphasis added] with a healthy lifestyle: the non-smoker, non-drinker must be healthy”.^5^ However, subsequent commentators (see e.g., Lucas & Lloyd, 2005), and Blaxter herself, remained skeptical that such responses should be interpreted literally. Instead, they argued that people likely substituted the difficult ontological question “What makes you call them healthy?” with the simpler causal question “What makes a person healthy?” Nevertheless, the idea that health is closely associated with a healthy lifestyle is also consistent with recent experimental philosophy findings that have challenged negativism about health. Varga and Latham (2024) found that laypeople and medical students judged that a person with a manageable disease leading an active lifestyle is both healthy and healthier than a person living a sedentary lifestyle who is disease-free.^6^ While this might indicate that health is closely associated with lifestyle matters rather than merely the absence of disease, Varga and Latham suggest that people might be responding to the risks of contracting a disease, rather than the disease itself.

Overall, the results of the corpus-linguistic study have shown a shift in people’s use of the term “healthy” toward lifestyle-related matters. Changes in the frequency of terms co-occurring with “healthy” indicate semantic shifts, i.e., suggest that the term “healthy” has either changed in meaning, or at least has a meaning that was not prevalent before the 80s. This interesting result warrants a more detailed exploration of the concept of health, which we undertake using two distinct methodologies. In Sect. 3, we carry out a series of semantic feature production tasks. Subsequently, in Sect. 4, we conduct a series of experimental vignette studies to further analyze the concept.

## Semantic Feature Production Tasks

Advocates of the classical theory of concepts attempt to determine the necessary and sufficient features of a concept. However, some features are distinguished by their salience—their ability to prominently stand out in our representation of the respective kind (see e.g., Fischer & Sytsma, 2021; Napolitano & Reuter, 2023; Reuter, 2024). For instance, when analyzing the concept of a wolf, we might identify features like < being a mammal > , < hunting in packs > , and < being dangerous for humans > . Being a mammal is a necessary property of wolves, marking < being a mammal > as a necessary feature of the concept. Next, many wolves hunt in packs, but some wolves are solitary (at least temporarily) and thus < hunting in packs > is a typical but not a necessary feature of the concept. Finally, wolf attacks on humans are rare but prominent given their significant impact on human perception and attention. Consequently, < being dangerous for humans > is a salient but far from necessary feature of the concept wolf.

One of the most commonly employed techniques for identifying the salient features of concepts is the semantic feature production task, or feature listing task. This approach has been extensively utilized in psychological research, with early instances noted by scholars such as Hampton (1979) and Barsalou (1983), and further discussed in detail by Machery (2023). In these tasks, as outlined in studies like McRae et al. (2005) and Beisbart and Reuter (2023), individuals are prompted to enumerate several attributes associated with a given word presented to them. In our studies, we used a similar prompt, asking participants: “Please name three features that you believe are characteristic of being *X*.”

In pilot studies, when participants were prompted to identify three characteristics associated with terms such as “healthy” and “good health”, the majority highlighted positive attributes, including “energy”, “fitness”, “happiness”, and “wellness”. Remarkably, only four out of 39 participants cited terms suggesting an understanding of health as the absence of disease. However, as it is common to associate health with positive qualities rather than the lack of negative ones, we proceeded to conduct semantic feature production tasks with the terms “unhealthy” and “sick”. These two tasks allow us to probe people’s understanding of health in light of the theories proposed. If health is the absence of disease or physical well-being, then participants should primarily state diseases or symptoms when being asked to state characteristic features of “unhealthy” and “sick”. If, however, a person’s lifestyle is not only conceived of as a causal factor for health but as a semantic feature of health, then participants should frequently name such lifestyle factors. 48 participants (12 men, 36 women, 0 non-binary, Mean age: 40.15 (SD = 13.82)) were recruited from Prolific and randomly assigned to either the “unhealthy” condition or the “sick” condition. The results from 24 participants assigned to “unhealthy” and another 24 to “sick” are summarized in Table 2 above and Table 3 below.

**Table 2:**
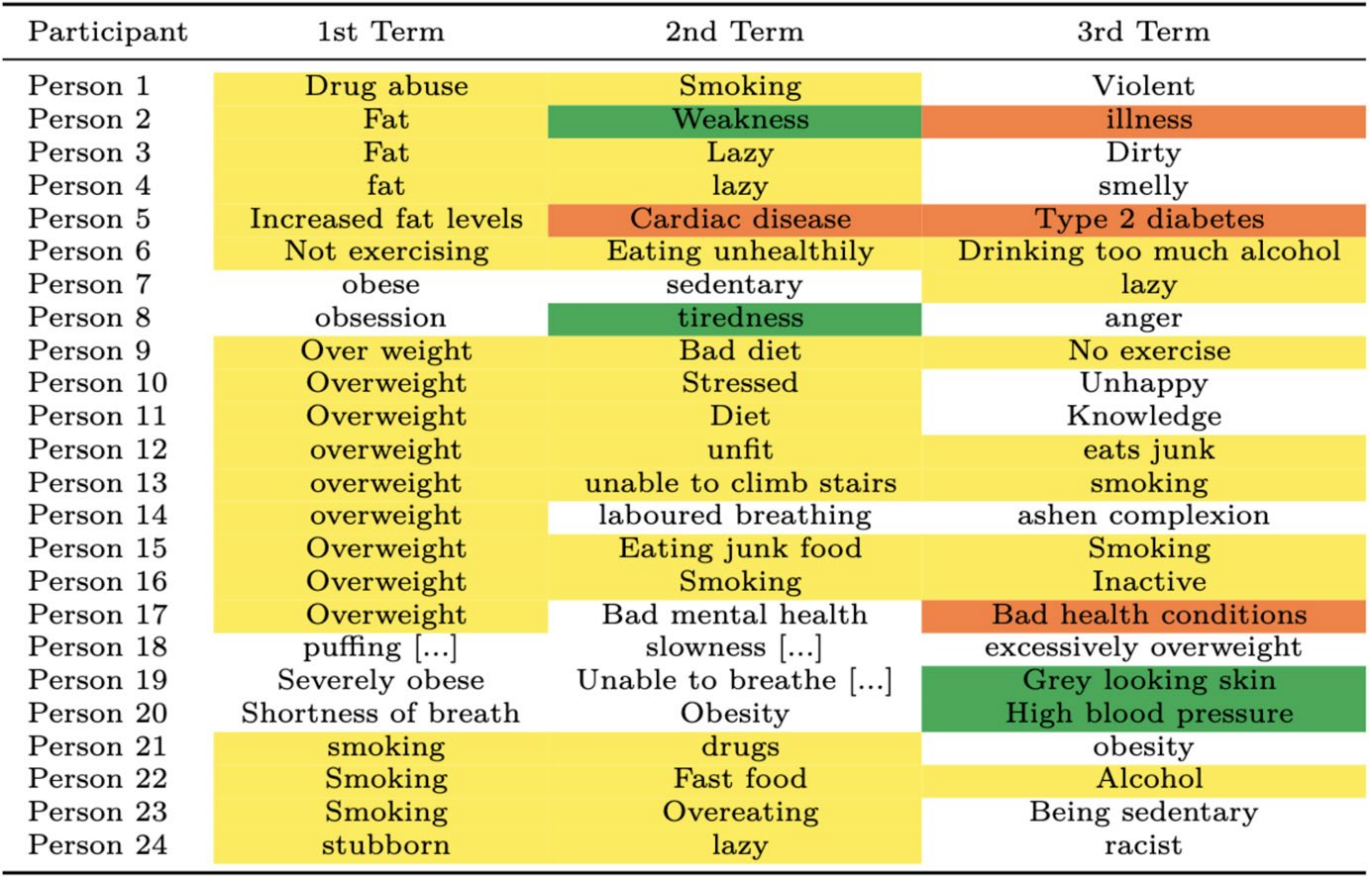
Responses of 24 participants to the term “unhealthy”

The authors used three categories to classify the responses: lifestyle responses (yellow), aspects of physical well-being (green), and disease-related responses (orange). The cells left uncolored indicate responses that were not categorized due to their ambiguity or unrelatedness

**Table 3:**
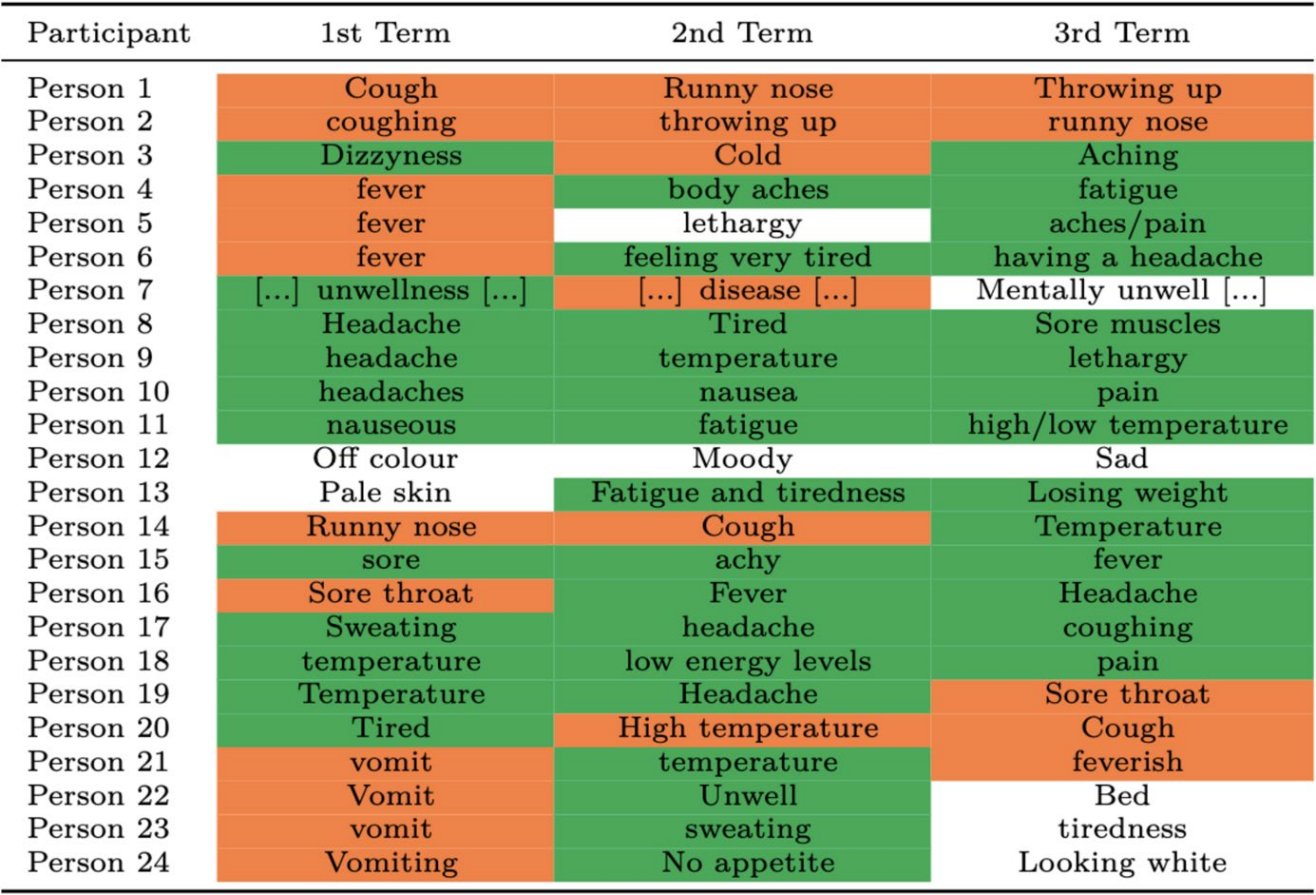
Responses of 24 participants to the term “sick”

The authors used three categories to classify the responses: lifestyle responses (yellow), aspects of physical well-being (green), and disease-related responses (orange). The cells left uncolored indicate responses that were not categorized due to their ambiguity or unrelatedness

### Results and Discussion

The outcomes of the Semantic Feature Production task for “unhealthy” challenge the notion that the concept of health is predominantly shaped by disease. Only three out of 24 participants (12.5%) mentioned terms directly related to diseases, and only four participants (16.6%) provided responses linking health with physical well-being. In stark contrast, a huge majority of participants (20 out of 24, 83.3%) wrote down terms associated with lifestyle factors.

The Semantic Feature Production task results for “sick” markedly diverge, with no references to lifestyle factors.^7^ Instead, nearly all participants report symptoms or diseases directly (such as cough, runny nose, cold, fever, disease, headache, tiredness, pain, etc.). This suggests that the concept of sickness aligns closely with traditional views found in the literature and illustrates a contrast between the notions of being sick and unhealthy. What is most salient to people about being unhealthy is not disease or discomfort, but lifestyle matters such as poor dietary habits, stress, lack of exercise, and using tobacco, alcohol, and drugs.

## Vignette Studies

The corpus data from Sect. 2 shows that there has been a marked shift in language use and potential mindset surrounding the concept of health. In the previous section, we then presented findings from Semantic Feature Production Tasks which revealed that when people consider the terms “healthy” and “unhealthy”, lifestyle-related matters, rather than symptoms or (the absence of) disease, are most salient. Furthermore, the salient features of “unhealthy” and “sick” are distinctly different: being sick is mainly linked to physical symptoms such as headaches, nausea, and fatigue, as well as common illness responses like coughing, fever, and a runny nose. In contrast, being unhealthy is associated primarily with lifestyle-related matters, including smoking, drinking alcohol, inactivity, poor diet, and being overweight.

However, to provide more solid evidence that lifestyle is central to the concept of health, the relevant factors need to be explicitly manipulated within an experimental framework. In this section, we describe the experimental studies we conducted to explore this relationship in detail. In Sect. 4.1, we describe the outcomes of a vignette study that illustrates the impact of lifestyle on people’s health assessments. Then, in Sect. 4.2, we describe the results of a second study that anticipates and addresses potential criticisms regarding the experimental approach in 4.1.

### Vignette Study 1: Methods and Hypotheses

In Vignette Study 1, we directly probe the effect of positive and negative lifestyle choices on people’s ratings of three terms. These terms are “sick”, “unhealthy”, and “healthy”. In both lifestyle conditions (positive / negative), participants are presented with a vignette about a person, called Erica, who is described as having had no diseases, and as having felt physically well during the last six months. The crucial third bit of information describes the person as having either made positive or negative lifestyle choices. The two vignettes read (“Positive” and “Negative” labels are just for illustration):

During the last six months, Erica has not had any diseases like infections, inflammations or bodily malfunctions.

During the last six months, Erica has also not experienced any symptoms like headaches, fever, or nausea.

*(Positive)* During the last six months, Erica has exercised regularly, slept sufficiently, ate properly, and did not smoke.

*(Negative)* During the last six months, Erica has not exercised, slept little, ate poorly, and smoked a lot.

Upon reading one of the two vignettes, participants were then asked to rate their agreement with the statement: “During the last six months, Erica was sick / unhealthy / healthy.” Participants answered on a 7-point Likert scale anchored at “1 = Totally Disagree” and “7 = Totally Agree.” We recruited N = 300 participants (197 female, 101 male, 2 non-binary, Mean age: 38.60 years (SD = 12.79)) from Prolific and randomly assigned them to one of the six conditions (positive—sick, positive—unhealthy, positive—healthy, negative sick, negative—unhealthy, negative—healthy). We introduced the term “sick” as a control to validate the findings of the Semantic Feature Production Task, which suggest that Erica should not be deemed sick, as she exhibited no diseases or symptoms impacting her physical well-being.

The following hypotheses were made and pre-registered https://osf.io/bzfkj on the Open Science Framework.

1. Hypothesis 1: There is a significant interaction between condition [positive vs. negative] and [sick vs. unhealthy/healthy]. Whereas participants’ agreement ratings for “sick” will be negative in both lifestyle conditions, i.e., participants do not consider Erica sick, participants’ agreement ratings for “healthy” and “unhealthy” will be positive in one and negative in the other lifestyle condition.
2. Hypothesis 2: For both the positive and the negative condition, ratings for sickness are below the midpoint.
3. Hypothesis 3: Whereas in the positive condition, ratings for unhealthy are below the midpoint (for healthy they are above the midpoint), in the negative condition, ratings for unhealthy are above the midpoint (for healthy they are below the midpoint).

After the main test question, participants are asked on a separate page: “Please tell us how relevant the following considerations were for you in your assessment of Erica’s health.” Participants rated the following three factors on a 7-point Likert scale ranging from “-3 = Not at All Relevant” to “3 = Totally Relevant”.

- The presence or absence of diseases or bodily dysfunctions.
- The presence or absence of physical symptoms.
- The lifestyle (i.e., sleep, food, exercise).

The purpose of this additional question was to validate our experimental design. Thus, in the case of the term “sick”, we expect participants to rate the presence/absence of disease and physical symptoms more highly than lifestyle. In contrast, we predict that participants will rate the lifestyle factor significantly more relevant compared to the other two factors for the terms “unhealthy”, and “healthy”.

#### Vignette Study 1: Results

**Fig. 2:**
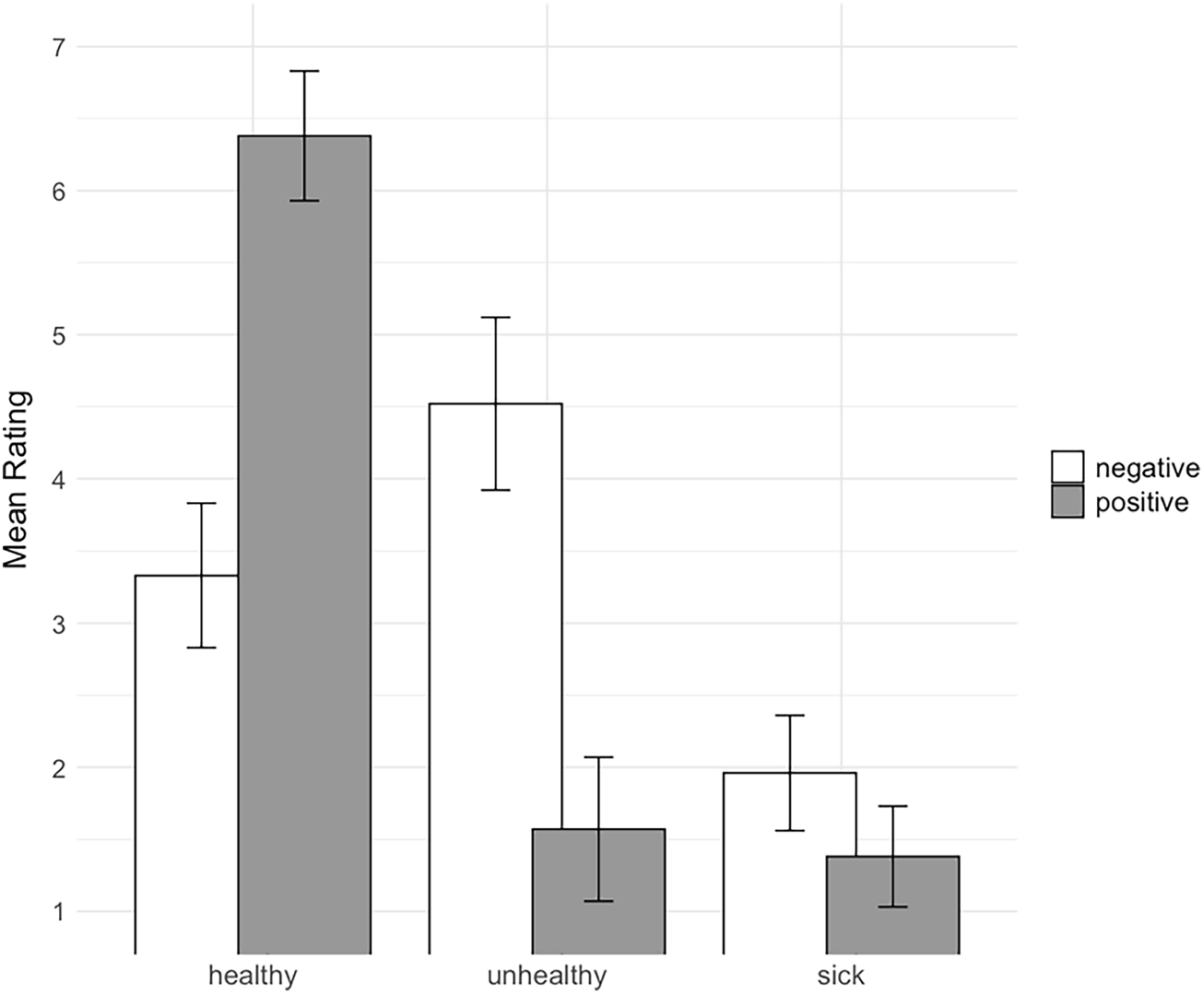
Results from Vignette Study 1, with error bars representing one standard deviation

Figure 2 shows the distribution of the responses for all three terms and both lifestyle conditions. Based on the assumption that “healthy” and “unhealthy” function as antonyms, we ran two 2 × 2 ANOVAS. As predicted (Hypothesis 1), the interaction between lifestyle (positive vs. negative) and term (sick vs. unhealthy) was significant (*F*(198) = 37.89, *p* < 0.001).

The average rating for the “sick” term was significantly lower than the midpoint of ‘4’ in both lifestyle conditions (positive—sick: M = 1.38, SD = 1.16, *t*(49) = − 16.00, *p* < 0.001; negative—sick: M = 1.96, SD = 1.36, *t*(50) = − 10.74, *p* < 0.001), which provides empirical support for Hypothesis 2. In contrast, in the negative lifestyle condition, the average ratings were significantly above the midpoint for “unhealthy” (M = 4.52, SD = 1.69, *t*(49) = 2.17, *p* = 0.017) and significantly below the midpoint for “healthy” (M = 3.33, SD = 1.55, *t*(50) = − 3.08, *p* = 0.002), supporting Hypothesis 3.^8^ This shows that participants believe that leading an unhealthy lifestyle is sufficient for being unhealthy. Comparing the results between healthy and unhealthy furthermore show that the terms function roughly as opposites, at least in this experimental setting. The results also confirm that *being sick* is considered to be a fundamentally different concept than *being unhealthy*.

**Fig. 3:**
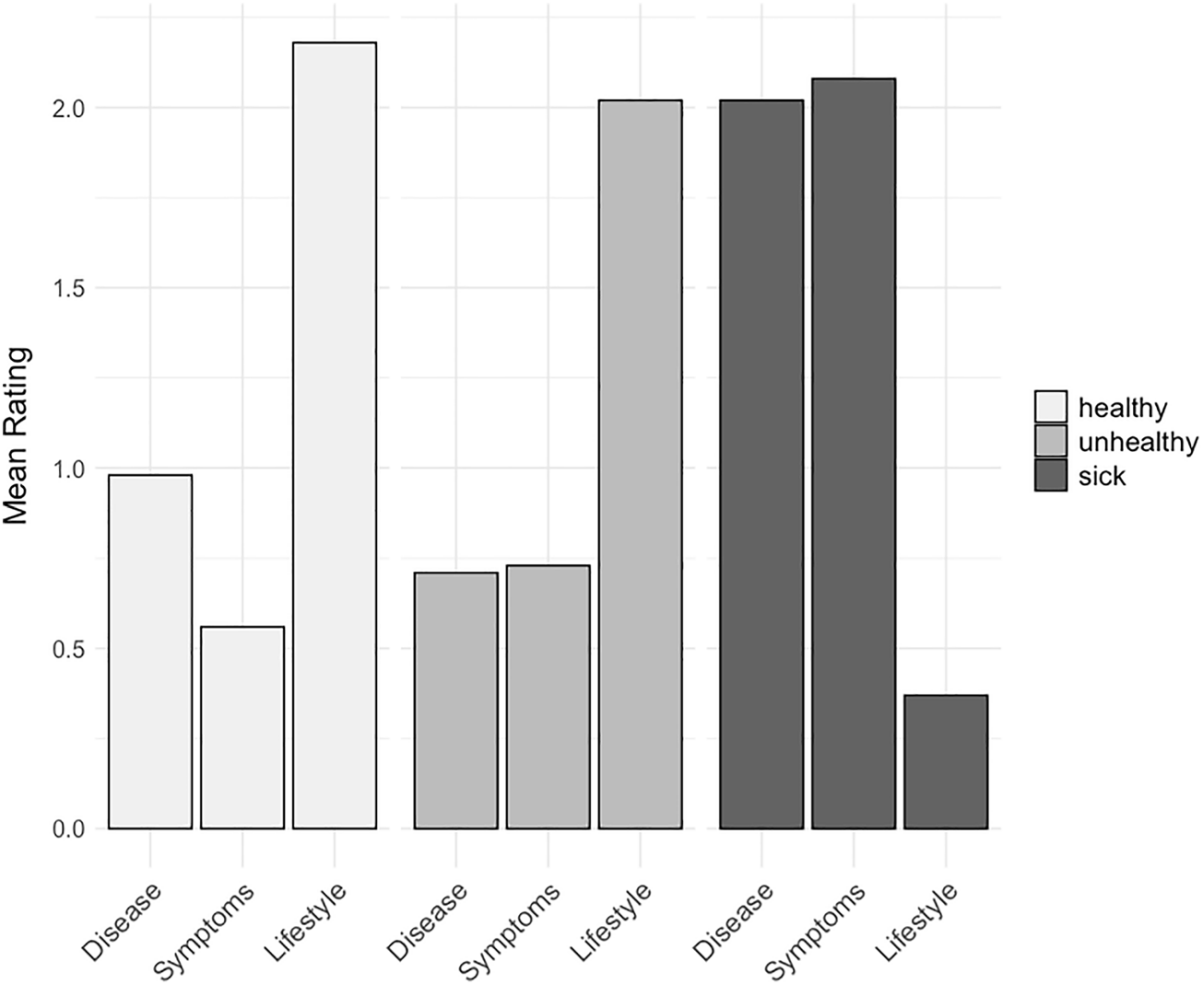
Relevance ratings of the three central factors in Vignette Study 1

We also analyzed the ratings to the three factors (second question), the results of which are displayed in Fig. 3. As predicted, the average rating for the lifestyle factor was significantly above the average ratings for the other two factors (*F*(2) = 15.79, *p* < 0.001 for negative—healthy; *F*(2) = 11.04, *p* < 0.001 for negative—unhealthy). In regards to the “sick” condition we predicted that the average rating for the lifestyle factor is significantly below the average ratings for the other two factors. This was also confirmed (*F*(2) = 12.87, *p* < 0.001 for negative—sick).

#### Vignette Study 1: Discussion and Objections

The findings from Vignette Study 1 indicate that participants deem a lifestyle characterized by minimal exercise, poor diet, and inadequate sleep as being *sufficient* grounds to judge an individual as being unhealthy. It is important to note, however, that it does not follow from these results that lifestyle is a necessary condition for such a judgment; indeed, positing it as a necessary condition would be highly implausible. Yet, to our knowledge, the significant influence of lifestyle on health judgments has not been extensively debated within the existing literature.

The vignette’s design might prompt some objections, which we anticipate will manifest primarily in three ways. First, the World Health Organization’s definition of health encompasses not just the physical but also the mental and social. The details provided in our vignette could be perceived as indicative of poor mental and social health. For instance, mentioning that Erica “slept little” might lead participants to infer negative aspects of her mental health contributing to her high stress and perhaps smoking and drinking habits. Therefore, to isolate lifestyle as the critical factor in determining Erica’s health status, we must refine the vignette’s language to preclude such an alternative explanation.

Second, the original vignette portrays Erica as someone who has not been diagnosed with any diseases and does not exhibit symptoms such as headaches, fever, or nausea. However, the absence of such symptoms does not conclusively indicate the absence of underlying diseases. Moreover, if participants interpreted the vignette from Erica’s point of view, they might consider that while Erica has not noticed any signs of disease, a physician might detect them. To provide epistemic certainty regarding Erica’s physical status, it is necessary to include an assessment from a third-party perspective, confirming her lack of disease and symptoms.

Third, in the original vignette, Erica’s lifestyle choices, characterized by smoking, insufficient sleep, and a poor diet over half a year, are certainly risk factors for serious diseases. It is reasonable then that participants might infer that Erica has a heightened risk (and/or disposition) to feel unwell or become ill. Therefore, when asked to assess whether Erica was unhealthy in the past six months, respondents may have interpreted the question as implying, “Over the last six months, did Erica exhibit a tendency to be ill or to develop health issues?” The structure of the first study’s vignette did not preclude such an interpretation by the participants.

### Vignette Study 2: Methods

In Vignette Study 1, our methodology extended beyond simply gathering participants’ agreement or disagreement with the statement about Erica’s health status. We also invited participants (in a pilot study) to elaborate on their judgments through a follow-up question. The entirety of these detailed responses is accessible in this open repository https://osf.io/c3ptm for review. Upon examination, one may observe that the majority of these elaborations do not robustly support the three counterarguments considered above. Yet, it is important to acknowledge that qualitative data alone might not completely or accurately capture the reasons behind the participants’ reactions. To address these objections more directly, we performed a second vignette study. In this subsequent study, we designed a series of vignettes, each aimed at confronting one of the three objections. The following vignettes were used:

**The Physical Health Case**

During the last six months, Erica has not had any diseases like infections, inflammations or bodily dysfunctions.

During the last six months, Erica has also not experienced any symptoms like headaches, fever, or nausea.

During the last six months, Erica has not exercised, slept little, ate poorly, and smoked a lot.

Please tell us how much you agree with the following question:

**During the last six months, Erica was physically healthy**

In the “Physical Health” scenario, we sought to be more explicit by asking participants to evaluate Erica’s “physical” health, addressing objection 1. We did this by including the term “physically” in the prompt. The rationale behind this approach was to isolate the concept of health as strictly physical, thereby differentiating it from mental or social considerations. If previously, participants were making inferences that included both physical and mental health when considering someone’s overall health, then incorporating the specifier “physical” in our query should sharpen the focus and significantly strengthen attributions related to physical health.

**The Epistemic Certainty Case**

During the last six months, Erica has not had any diseases like infections, inflammations or bodily dysfunctions. In fact, Erica’s physician, who has continuously monitored Erica during the last six months, informs her that all of her test results have been normal during this period (blood pressure, cholesterol, triglycerides, body mass index, and so on).

During the last six months, Erica has also not experienced any symptoms like headaches, fever, or nausea.

During the last six months, Erica has not exercised, slept little, ate poorly, and smoked a lot.

Please tell us how much you agree with the following question:

During the last six months, Erica was healthy.

To address the issue of epistemic certainty (objection 2), we incorporated details about a comprehensive health check conducted by a physician. The purpose of this addition was to counteract concerns that participants might harbor about potential undetected diseases. These changes should prevent participants from inferring that the mere absence of noticeable symptoms, as perceived from Erica’s perspective in the original vignette, does not necessarily preclude the existence of underlying diseases.

**The Genetic Advantage Case**

During the last six months, Erica has not had any diseases like infections, inflammations or bodily dysfunctions.

During the last six months, Erica has also not experienced any symptoms like headaches, fever, or nausea.

During the last six months, Erica has not exercised, slept little, ate poorly, and smoked a lot.

Erica, though, is naturally immune to the usual problems linked to little exercise, not enough sleep, bad eating habits, and smoking, thanks to her genes. This means she is guaranteed to never suffer from these problems.

Please tell us how much you agree with the following statement:

During the last six months, Erica was healthy.

The vignette above as well as the following vignette were used to address objection 3. In the “Genetic Advantage” scenario, we specify that Erica possesses a natural immunity to the problems typically associated with her poor lifestyle choices. If participants in the original vignette were inclined to judge based on Erica’s susceptibility to diseases, then this addition should counteract that assumption. The intent is to explore whether these changes in future risk to develop diseases alter people’s judgments regarding Erica’s health.

**The Change of Lifestyle Case**

It is now April 2024.

During the last six months of 2023, Erica has not had any diseases like infections, inflammations or bodily dysfunctions.

During the last six months of 2023, Erica has also not experienced any symptoms like headaches, fever, or nausea.

During the last six months of 2023, Erica has not exercised, slept little, ate poorly, and smoked a lot.

Beginning in 2024, Erica made a series of changes in her life: she begun exercising regularly, improved her sleep and diet, and quit smoking.

Please tell us how much you agree with the following statement:

During the last six months of 2023, Erica was healthy.

In the final scenario, termed the Change of Lifestyle case, we anchored the narrative by setting a precise temporal context (April 2024). We initially provided participants with the same details as in the initial vignette, but then informed them that starting from 2024, Erica undertook significant lifestyle changes, including adopting a regular exercise routine, ceasing to smoke, and improving her sleep patterns and dietary habits.

We pre-registered https://osf.io/w5t9v the following three hypotheses to examine the validity of the objections through a three-tiered testing approach, each varying in stringency:

1. Hypothesis 1: If one or more objections comprehensively account for the observed health ratings, the health ratings under these objection conditions will not be significantly lower than those seen in the positive lifestyle baseline condition.
2. Hypothesis 2: If one or more objections can reverse the negative health ratings, then the health ratings under these objection conditions will be significantly higher than the midpoint rating of “4”.
3. Hypothesis 3: If one or more objections fail to address the negative health impacts attributed to lifestyle factors, the health ratings under these objection conditions will not be significantly higher than those recorded in the negative lifestyle baseline.

#### Vignette Study 2: Results

**Fig. 4:**
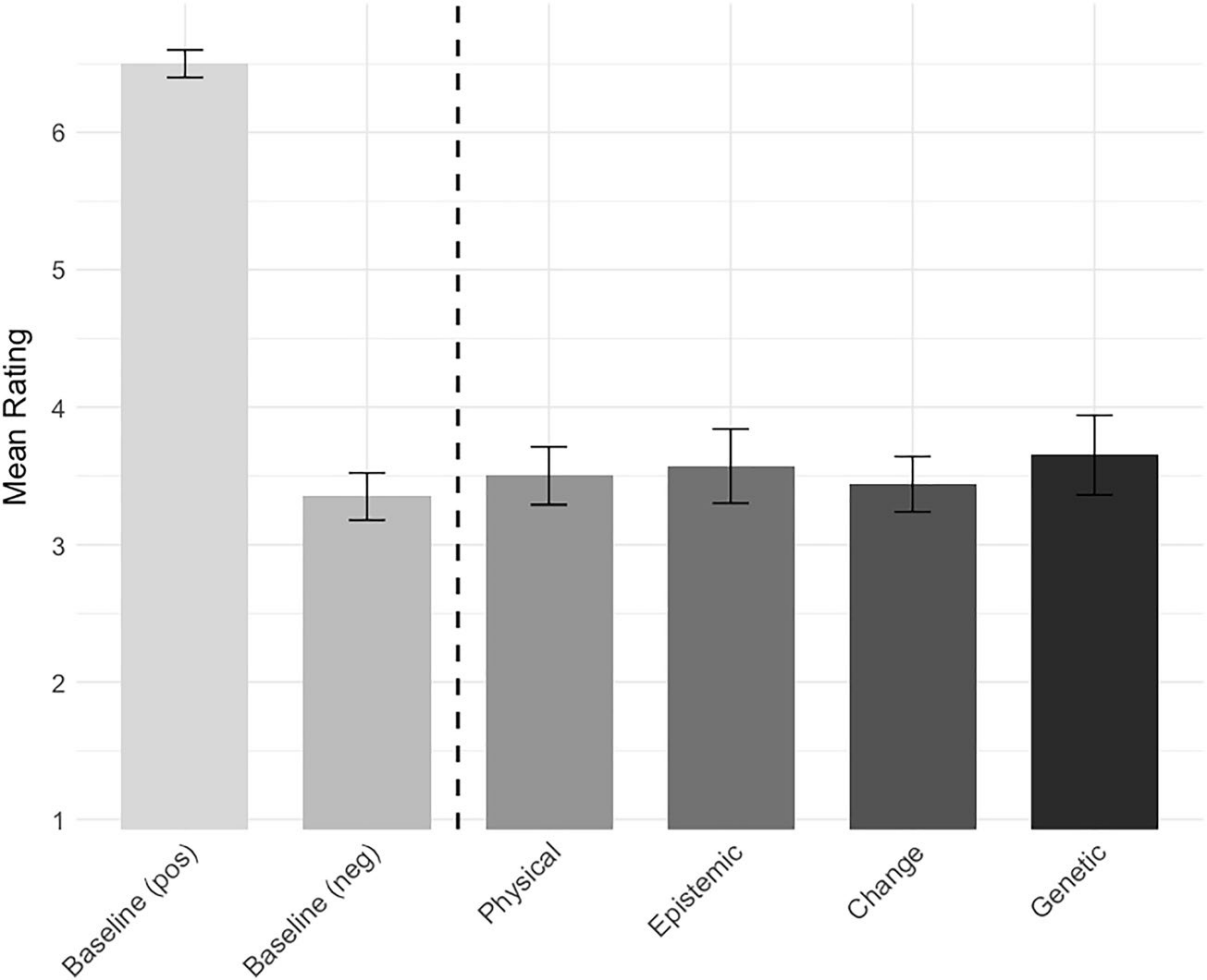
Results from Vignette Study 2. Error bars indicate standard error around the means

301 participants (118 male, 182 female, 1 non-binary, Mean age: 41.33 years) were recruited for Study 2 using Prolific and randomly assigned to one of six conditions. Figure 4 presents the average ratings for the two baseline conditions alongside the four objection conditions. The mean rating for the positive baseline condition is significantly above the midpoint of 4 (M = 6.50, SD = 0.74, *t*(49) = 24.04, *p* < 0.001), indicating a strong positive assessment, whereas the mean for the negative baseline condition falls significantly below this midpoint (M = 3.35, SD = 1.20, *t*(50) = − 3.86, *p* < 0.001), reflecting a negative assessment. These results align closely with those of Study 1, effectively replicating its findings.

For all four objection conditions, the average ratings are below the midpoint of 4. This outcome allows us to conclusively reject Hypotheses 1 and 2. To further explore Hypothesis 3, we conducted significance tests comparing each objection condition against the negative baseline. The results yielded the following t-statistics and *p *values: Physical (M = 3.50, SD = 1.50) vs. Baseline (*t* = − 0.54, *p* = 0.59), Nowbetter (M = 3.44, SD = 1.40) vs. Baseline (*t* = − 0.34, *p* = 0.74), Physician (M = 3.57, SD = 1.86) vs. Baseline (*t* = − 0.70, *p* = 0.49), and Genetic (M = 3.65, SD = 2.06) vs. Baseline (*t* = − 0.88, *p* = 0.38). All p-values exceeded 0.05, indicating no statistically significant differences between the objection conditions and the negative baseline condition.

**Fig. 5:**
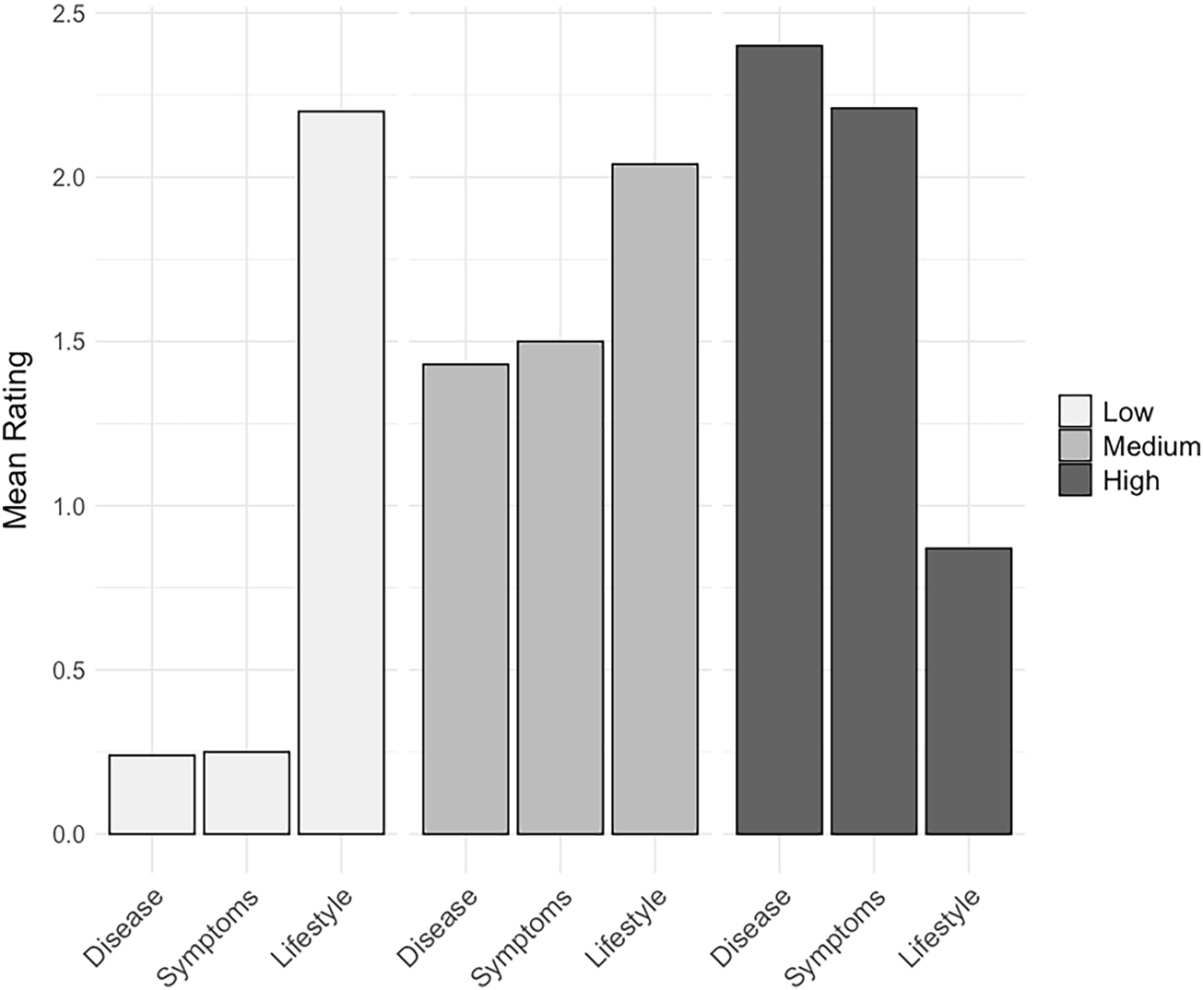
Relevance ratings of the three central factors in Vignette Study 2

In addition to health ratings, participants were asked to evaluate the relevance of three key factors—disease presence, symptom presence, and lifestyle choices— in their assessments of Erica’s health (see also Vignette Study 1). Figure 5 displays the mean relevance ratings categorized by participants’ health assessments: those who rated Erica’s health below ‘4’ (Low), exactly ‘4’ (Medium), and above ‘4’ (High).

#### Vignette Study 2: Discussion

Given the outcomes of Vignette Study 2, we can reject all three hypotheses, reinforcing the robustness of our original findings. These findings compellingly demonstrate the significant impact of lifestyle choices on health judgments. Furthermore, our results have proven to be highly resilient across four different methodological modifications.

Based on the findings from Vignette Study 2, we can refute three potential criticisms of Study 1’s results. These criticisms concerned different interpretations of health within the study context. Specifically, we addressed: (a) the interpretation of health as (or including the) mental or social by including the term “physical” to clarify that the original prompt pertained to physical health. (b) The suspicion of undiagnosed diseases by including a physical check-up to counter objections that participants may doubt Erica’s health status due to potential undetected diseases. (c) The potential for future disease development by including two different conditions designed to prevent a dispositional interpretation of the term “healthy”.

Despite the findings from Vignette Study 1 and 2, it is important to acknowledge that a substantial minority does not view poor lifestyle choices as solely indicative of poor health. Combining data from the negative baseline and four objection conditions, 62 out of 252 participants (24.6%) rated “5” or higher, suggesting they considered the individual, Erica, to be healthy based solely on the absence of disease and symptoms.

Additionally, 49 participants (19.4%) assigned a neutral rating of “4”, indicating that they do not regard lifestyle choices as the definitive factor in health assessments. However, a significant majority of 140 participants still perceived Erica as unhealthy.

One helpful referee asks whether it is possible that people could be muddling the distinction between the specific state of an organism and a factor that reliably contributes to such a state being the case. People might be treating each of the cases we examined as asking whether Erica *lives* healthily, but if they thought what was being asked was whether Erica was in a *state* of good physical health, then they would be expected to reliably agree. To address this concern, we ran an additional study.^9^ We reran the healthy and unhealthy conditions from Study 1 and Study 2, but this time asked either (1) “During the last six months, Erica was in good physical health” or (2) “During the last six months, Erica was in poor physical health.” If the referee’s concern is right, then we would predict higher mean ratings when participants are asked (1), and lower mean ratings when participants are asked (2). However, that is not what we found. In fact, mean ratings appeared higher in the poor physical health condition (M = 4.18, SD = 1.72) than the good physical condition (M = 3.68, SD = 1.49), a difference which was significant, *t*(74.29) = − 1.39, *p* = 0.017. Importantly, ratings when asked whether Erica is in good physical health were not significantly different to Study 2 when asked whether Erica is physically healthy (M = 3.50).

## General Discussion

In this paper we have provided new empirical data from corpus linguistics, semantic feature production tasks, and vignette studies that speak to the relational issue in the philosophy of medicine. Recall that the relational issue concerns what the conceptual relationship is between the concepts of health and disease. Negativism claims that health is just the absence of disease, whereas positivism claims that health is the presence of some further positive state or capacity. Each of the lines of empirical data that we have examined converge and suggest that negativism is not true of most people’s ordinary concept of health.

Our corpus linguistic study provides an initial indication that what matters most for contemporary health judgments are certain patterns of choices and behavior that have become associated with a “healthy lifestyle”. As we noted in the discussion of those results, we think that this contemporary shift likely reflects heightened health awareness and the promotion of preventive medicine through public health efforts, as emphasized by organizations such as the WHO. Consequently, “healthy” is not the absence of disease, but instead encompasses behaviors commonly recognized in both the academic literature and popular discourse as being elements of a healthy lifestyle. The corpus linguistic results were further corroborated by the results of a semantic feature production task. This task revealed that what is most salient to people about being “unhealthy” is not the presence of disease, but rather certain choices and behaviors such as smoking, excessive alcohol consumption, inadequate sleep, and poor diet. In response to an “unhealthy” prompt, only a small fraction (12.5%) of the reported responses directly related to diseases. Instead, the vast majority (83.3%) of people reported lifestyle-related terms.

While corpus data and semantic feature tasks provide insight into language use and associations, on their own they do not tell us whether people’s health judgements depend on people’s lifestyles. Testing that was the role of our vignette studies. The results from Vignette Study 1 suggest that leading a healthy lifestyle is, overall, necessary to be deemed healthy, whereas leading an unhealthy lifestyle is, overall, sufficient for being deemed unhealthy. Importantly, when participants were asked what the most important factor was for determining their “healthy” and “unhealthy” judgments, lifestyle was by far the most dominant factor. Vignette Study 2 replicated the results of Study 1 and ruled out several potential objections that might have explained away our results in a manner consistent with negativism. These objections were related to (a) the interpretation of the health prompt, (b) potential undiagnosed diseases, and (c) the likelihood of future disease.

Our findings suggest then that most people do not subscribe to a negativist account of health—though it is worth acknowledging that roughly a quarter of our participants gave answers which are consistent with negativism. Of course, this leaves open the possibility of *restricted* negativism. Restricted negativism is the view that negativism is at least true of the concept of health in theoretical medicine (e.g., Boorse, 1997, 2014). One possibility then, is that those people who respond consistently with negativism do so as a result of borrowing this scientific concept. Note, however, that many scholars in the philosophy of medicine hold that *unrestricted* negativism is true. As such, our studies challenge this widely-held view in philosophy of medicine.

### Implications for Defining Health

If most people’s health judgments are not tracking the presence or absence of disease, then what else could they be tracking? The alternative position is positivism, according to which health tracks the presence of some further positive state or capacity of the person. Let us consider two different versions. First, people’s health judgments might be tracking some further positive state or capacity that is defined in disease-related terms. For instance, physical resilience is the capacity to achieve, retain, and/or regain normal physical function after a disease. Second, people’s health judgments might be tracking some further positive state or capacity which is defined independent of disease. For instance, if health judgments are ultimately tracking a state of well-being, then depending on how one characterizes well-being, it is possible that health comes entirely apart from disease-related matters. In each case, people’s health judgments could be tracking such states or capacities, with lifestyle information being used as evidence that the evaluation target possesses them.

Let us explore these two options. First, even if our results indicate that negativism is not true of the concept of health, positivism might ultimately be understood in disease-related terms. For instance, the suggestion by Varga and Latham (2024) that someone is healthy only if they are not at-risk from disease could easily be given a positive spin: Someone is healthy only if they are physically resilient. This lack of risk might well be associated with a healthy lifestyle, which is why such a lifestyle is deemed necessary for health by people. If health is understood in such a disease-related manner, then the resulting account still looks like a negative proposal: health requires leading a lifestyle which prevents disease development and slows disease progression or, as positivism would describe it, by inculcating certain positive traits and capacities such as resilience.

Although we cannot entirely dismiss such a disease-centered theory of health, we found no supporting evidence for this view. In fact, we found three bits of empirical evidence that speak against this proposal. First, if it were right, then we would expect participants to highlight disease-related factors in the semantic feature production task. This is not what we found. Second, we would expect people to report disease symptoms as being the most or at least one important factor for determining “healthy” and “unhealthy” judgments (in both Vignette studies). Again, this was not the case in our findings. Third, recall the positive genetic condition in Study 2. In that case, Erica was engaged in a negative lifestyle, but due to her constitution, she was not at risk of any of the usual disease related consequences. Despite this, people still judged Erica to be unhealthy. While there might be some ways in which our data can be reinterpreted to make sense of Option 1,^10^ a disease-dependent theory looks more and more unlikely to be true of laypeople’s understanding of health. Nonetheless, it is, of course, possible that Option 1 holds true for those participants (roughly 25%) who responded in line with standard negativism.

How about Option 2? Health judgments track some positive state or capacity which is defined independent of disease. When we consider our results then there is an open question regarding what it is that positive and negative lifestyles are being associated with such that Erica is being correctly judged to be “healthy” or “unhealthy”. For example, imagine as a toy example that health tracks well-being and that well-being is best understood in terms of having more of your desires satisfied than not. Thus, one reason that people might judge that Erica in the positive lifestyle case is healthy and Erica in the negative lifestyle case is unhealthy might be that they attribute to Erica a standing desire for a positive lifestyle and not a negative lifestyle. Of course, there is nothing special about this toy case and we might construct different stories about how the choices and behaviours of a healthy and unhealthy lifestyle might be associated with one’s preferred positive states or abilities in the literature.

While our results are compatible with Option 2, some positive states, especially those that the WHO has focused on, seem to be ruled out by our studies. Note that the WHO talks about “physical well-being”, which surely is a vague term to begin with. Nonetheless, in the vignettes we specified that Erica does not experience any headaches, fever, or nausea, the corollary which would be something like “feeling physically fine”. From this we can at least infer that any positive state still compatible with our results is unlikely to be characterized in disease-related terms. Further studies are required to investigate which states and capacities, posited by positivists, people are tracking with their health judgments.

At the beginning of this paper, we noted a further option which is consistent with the current results. That is, all that matters to people for judging a person to be healthy is that person’s lifestyle choices and behaviours. And this would be the case, regardless of whether they are associated with some further positive states or capability. Such a view would be in line with Blaxter’s interpretation (which she does not endorse) of some people’s responses in the health and lifestyle survey. If health is simply identical with a healthy lifestyle, then our results would not only put pressure on negativism but also on its main contender positivism.

Admittedly, such a view seems just false. A person who makes perfect lifestyle choices but is suffering from a painful disease is likely not to be judged healthy. That said, perhaps people do make an important distinction between (a) a concept of health that refers to healthy lifestyle choices and (b) a concept of health that refers to being in a state of health. Our results on “unhealthy” and “sick” clearly show that people make an important distinction between health and sickness. It just so happens that under ordinary circumstances, people tend to overwhelmingly emphasize (a) over (b) in their health talk. However, as the case of a perfect lifestyle with a painful disease shows, there are contexts in which we can be brought to shift the emphasis to (b). Understanding health thus requires considering both lifestyle practices and the physiological state of the individual.

### Further Implications

The strong association between health and lifestyle can have practical consequences outside of philosophical debates. For instance, if lifestyle is understood in terms of a person’s choices and behaviour, then it is natural to assume that lifestyle is under a person’s direct control. But that would be too simplistic and would fail to acknowledge the role that the environment plays in shaping choices and behaviours. The failure to recognize the significant role played by the environment very likely contributes to the stigmatization of certain individuals by attributing (perhaps moral) responsibility for their health that is unwarranted.

It is much easier to choose to eat a healthy diet when your friends and family do, when you have been educated on correct dietary choices, when good and fresh food is readily available and affordable, and so on. Of course, people in environments without any of those things are (perhaps) still free to choose to eat a healthy diet, but their choice is much more costly to make. Failure to recognize that not everyone’s choices and behaviours are the same can result in people being unfairly disparaged.

Finally, our findings could have practical impacts on healthcare delivery and public understanding. The way that people understand health and being healthy seems to partially align with the concept of health used in public health contexts, where the primary goal is the adoption of healthy lifestyles. Thus, when public health interventions are run aimed at increasing health, the measure of success is typically the adoption of those lifestyles. However, lifestyle in public health is often understood in disease-related terms (see previous section). So, while there might be agreement regarding paradigmatic choices and behaviours that constitute a positive or negative lifestyle, what makes them healthy, and to what extent, might be completely different. Of course, more research is required to determine if this is a significant issue, but it is reasonable to suspect that such a discrepancy could lead to communication failures. Patients might feel that their broader health concerns are being ignored if doctors and public health professionals ultimately concentrate on disease-related matters, leading to misunderstandings about goals, mismatched expectations, and dissatisfaction with services. Consequently, patients may expect a more holistic approach and may desire interventions that include both medical treatment and positive lifestyle guidance.

Before concluding it is worth noting several limitations in our research that future research should seek to address. First, under the label of “health-related lifestyle” we only examined paradigmatic choices and behavioral patterns such as smoking, dietary habits, sleep habits, and physical activity. It is important to note, however, that the concept of “health-related lifestyle” is not entirely clear. For example, it is entirely unclear why other choices and behavioral patterns such as travel habits and social interactions are less emphasized or outright excluded, despite their substantial relevance for health. Second, the use of vignettes containing hypothetical scenarios, while controlled, introduces a level of abstraction that might not accurately reflect people’s actual judgments. Second, the focus on an English corpus and English-speakers in our studies may limit the generalizability of our findings across different cultural contexts and future research should include more diverse populations. Third, to assess any possible implications, more studies are required to know whether the concepts of health and disease described here align with those used by health professionals. Finally, although we find that most people lean towards a positivist view of health rather than a negativist one, it is important to note that the results are consistent with the possibility of health being a singular, evolving, and multi-aspect concept, but also with the possibility that there are multiple, coexisting concepts.

## Conclusion

Our research employed a multi-method approach that includes corpus linguistics, semantic feature production tasks, and vignette studies to better understand the concept of health. This multi-methodological strategy avoids many of the traditional methodological confines and provides both a clearer understanding of the concept of health and how it evolved.

Our main finding about the link of health to certain health-related behavioral patterns has implications for the relational issue in the philosophical-medical debate on health and disease. Our results indicate that most people do not adhere to a negativist perspective on health, but are instead positivists about health. Further research, however, is required to investigate exactly what states and capacities people’s health judgments are tracking and whether these are the same as those posited by positive theorists in the medical literature.

This issue of health has consequences beyond philosophical debates and has implications for health communication and policy development. First, perceiving health as merely lifestyle choices, without considering the influence of factors beyond an individual’s control, can grossly oversimplify health understanding and foster stigmatization. Second, the disparity between the holistic understanding of health that includes lifestyle and the clinical focus on disease symptoms could lead to communication gaps. Third, the distinction between being “unhealthy” and “sick” should be taken into consideration in health communication strategies and targeted health interventions.

## Footnotes

1 For instance, Cooper (2020, 144) notes that "In so far as philosophers seeking to develop accounts of disorder engage in descriptive conceptual analysis, accounts must broadly fit with intuitions about the disorder status of particular conditions."

2 Throughout this paper we will talk in terms of what are the necessary and sufficient features of health, as is common (though not ubiquitous) in the philosophy of medicine literature. None of our results which we will report here adjudicate between what the correct theory of concepts is and readers should feel free to parse these results according to their own preferred views. For instance, if we say that our results suggest that a particular feature is sufficient to be judged to be unhealthy, then you might also possibly read our results as suggesting that a particular feature is a part of the prototype of unhealthy, or a highly weighted feature of unhealthy, and so on.

3 Corpus linguistic investigations encompass studies into the use of the terms “pain” (Reuter, 2011), “discrimination” (Willlemsen et al., forthcoming), “causation” and “responsibility” (Sytsma et al., 2019), “reasonable” (Baumgartner & Kneer, 2023), “conspiracy theory” (Reuter & Baumgartner, 2024), and many more.

4 This shift might be attributed to the heightened prevalence of terms such as “lifestyle”, “eating”, “diet”, “food”, and “weight” in recent discourse. If these terms have become more common over the past four decades, then the use of “healthy” may not have fundamentally changed; instead, what has evolved is our application of these lifestyle-related terms. Upon examining the frequency of terms like “eating”, “diet”, “food”, and “weight”, we find no such shift but rather observe a relatively stable use across the nineteenth and twentieth century. The notable exception is “lifestyle”, a term whose usage has indeed surged since the 1980s.

5 The timing of the Health and Lifestyle Survey data aligns intriguingly with our corpus data. Our corpus analysis indicates that discussions about healthy lifestyles began to emerge in the early 1980s. The survey, conducted in the late 1980s, reveals that younger adults, in particular, were most influenced by lifestyle choices in their understanding of health. Therefore, it is not surprising that younger adults would be most strongly influenced by lifestyle choices in their understanding of health.

6 Similarly, a 2022 survey (https://www.bfs.admin.ch/news/en/2023-0115) revealed that 85% of Swiss residents aged 15 and older reported feeling in good health, even though over a third were managing long-term health issues, suggesting that good health and chronic disease can coexist.

7 Pearson *χ*^2^ tests indicated significant differences between the “unhealthy” and “sick” condition: lifestyle responses (*χ*^2^ = 34*.*29, *p* < *.*001), well-being responses (*χ*^2^ = 31*.*45, *p* < *.*001), and disease responses (*χ*^2^ = 12*.*80, *p* < *.*001).

8 The results for positive—unhealthy were: M = 1.357 SD = 1.20, *t*(50) = -14.42, p < 0.001; and for positive—healthy they were: M = 6.38, SD = 0.78, *t*(49) = 21.59, *p* < 0.001.

9 There were 78 participants (54 women, 23 men, 1 non-binary. Mean age: 35.02). Data for this further study can be found on the Open Science Framework: https://osf.io/c3ptm.

10 For instance, a defender of Option 1 might point out that while Erica’s constitution prevents the usual disease related consequences of her unhealthy lifestyle, it does not prevent all of them, and so people are right to judge that she is unhealthy. Alternatively, they might argue that what matters is not the consequences of a lifestyle for any given individual, but the typical consequences of that lifestyle for people. Erica then is unhealthy, because the lifestyle that she is engaged in typically brings about diseases in people, even if it does not in her.
